# Case Report: A Pancreatic Ductal Adenocarcinoma Patient With Concurrent Targetable Somatic Novel KANK1-ALK, UPP2-NTRK3 Fusion, and Pathogenetic Germline BRCA Mutation

**DOI:** 10.3389/fonc.2021.757965

**Published:** 2021-10-04

**Authors:** Fan Meng, Le Lu, Yuan Tan, Qianqian Duan, Hongwei Lu

**Affiliations:** ^1^ Department of General Surgery, The Second Affiliated Hospital of Xi’an Jiaotong University, Xi’an, China; ^2^ The Medical Department, Jiangsu Simcere Diagnostics Co., Ltd., Nanjing, China; ^3^ Nanjing Simcere Medical Laboratory Science Co., Ltd., Nanjing, China; ^4^ The State Key Lab of Translational Medicine and Innovative Drug Development, Jiangsu Simcere Diagnostics Co., Ltd., Nanjing, China

**Keywords:** PDAC, KANK1-ALK, UPP2-NTRK3, pathogenetic BRCA1 mutation, next-generation sequencing

## Abstract

Pancreatic ductal adenocarcinoma (PDAC) is presently one of the cancers with the worst survival rates. The current treatment options for PDAC are relatively scarce due to insufficient understanding of molecular characteristics and subtypes of PDAC. Based on next-generation sequencing (NGS), we firstly presented a case about a KRAS wild-type pancreatic ductal adenocarcinoma patient harboring a concurrent targetable rare somatic novel KANK1-ALK, UPP2-NTRK3 fusion, and pathogenetic germline BRCA mutation. These two novel fusion statuses were assayed by immunohistochemistry (IHC) and fluorescent *in situ* hybridization (FISH). Our findings demonstrated that comprehensive and systematic screening of PDAC for actionable genomic alteration may substantially improve the therapeutic prospects for a sizeable fraction of patients with PDAC. To improve the management of PDAC in an era of precision medicine, it is important to identify ALK or NTRK fusion-positive and pathogenic germline mutation subsets of patients who can benefit from targeted therapies.

## Introduction

Pancreatic ductal adenocarcinoma (PDAC) is one of the deadliest malignancies with a high mortality rate and poor survival across all stages (1). Approximately 88%–95% of pancreatic adenocarcinomas harbor KRAS driver mutations. There is only sotorasib as a KRAS-G12C inhibitor-targeted therapies for nonsmall-cell lung cancer (NSCLC)—approved by the US Food and Drug Administration (FDA), but high-quality evidence is lacking in pancreatic cancer (2). While in KRAS wild-type tumors, alternate oncogenic drivers have been identified, including BRAF, ROS, NRG1, GNAS, CTNNB1, and anaplastic lymphoma kinase (ALK) gene fusions (3, 4). Since ALK gene fusion was first identified in pancreatic cancer in 2017, so far, only sporadic ALK translocation cases have been reported in PDAC (5, 6). Also, relevantly, few of neurotrophic tropomyosin receptor kinase (NTRK) fusion PDAC patients have been reported sensitive to NTRK inhibitors (7, 8). Besides that, with the development of precision medicine, targeted treatment of pancreatic cancer has achieved some success. For instance, more recently, the FDA approved the PARP inhibitor olaparib for use in patients with PDAC who have a germline mutation in either BRCA1 or BRCA2 (9). Since a few numbers of PDAC patients harbor target driver gene, the form of mutation, the efficacy of targeted therapy, and prognosis need more attention and research (10).

In this case, we first reported targeted comutation of somatic novel KANK1-ALK, UPP2 intergenic NTRK3 fusion, and pathogenetic germline BRCA1 mutation in a pancreatic ductal adenocarcinoma patient. As far as we know, both ALK and NTRK mutations have only less than 1% mutation frequency in PDAC, while concomitant multiple targetable variations have not been reported.

## Background

**Figure 1:**
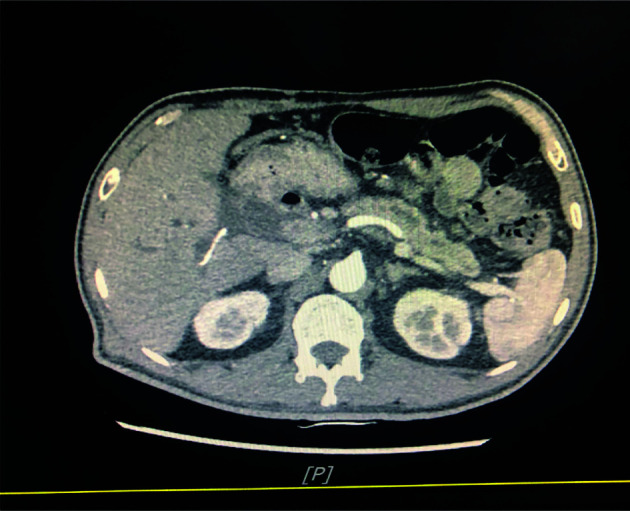
Computed tomography shows pancreatic lesion before the surgery.

A 54-year-old man was admitted to our hospital because of sclera and skin yellowing and abdominal pain. The patient has no history of smoking and drinks alcohol occasionally. He has no history of infectious diseases such as hepatitis, tuberculosis, and malaria and has no history of hypertension. Furthermore, his mother and aunt had a history of ovarian cancer. Examination after admission showed a significant increase in serum biomarkers, including carbohydrate antigen CA199 and carbohydrate antigen CEA. The abdominal computerized tomography (CT) showed enlargement of the head of the pancreas with double-duct disease (**Figure 1**). Pathological examination demonstrated a ductal adenocarcinoma of the pancreas. Pancreaticoduodenectomy (Whipple technology) was performed, and albumin-bound paclitaxel 125 mg/m^2^ qw3/4 combined gemcitabine 1,000 mg/m2 qw3/4 × 6 cycles were given as postoperative adjuvant therapy.

**Figure 2:**
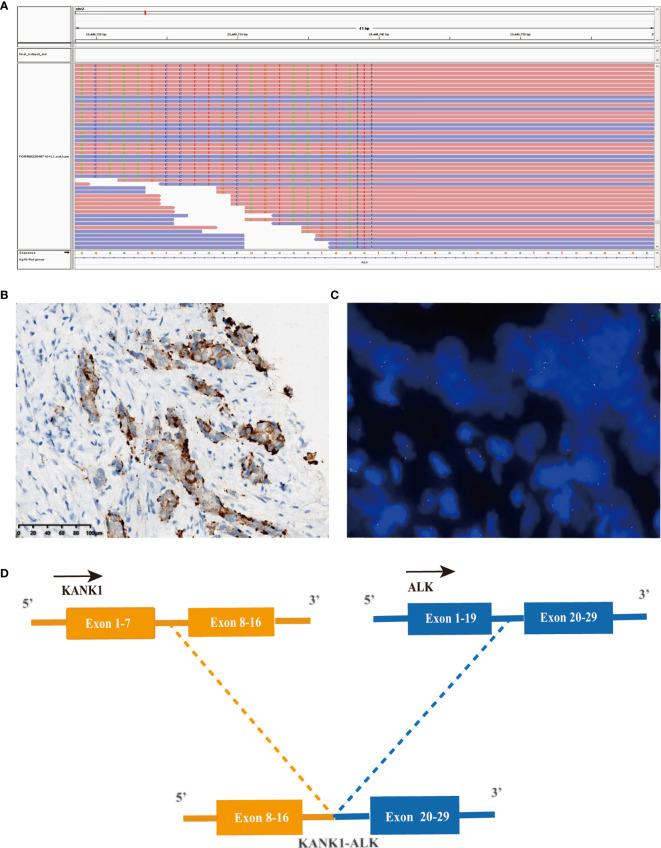
A novel KANK1-ALK fusion was discovered in a PDCA patient. **(A)** Sequencing reads of ALK is shown by the Integrative Genomics Viewer. **(B)** Immunohistochemistry staining indicated expression of ALK, the specimen was stained by IHC with the anti-ALK (D5F3) primary antibody. **(C)** A split signal was observed with a frequency of 18% in the fluorescence *in situ* hybridization image using ZytoLight ALK Break Apart FISH Probe. **(D)** Illustration of KANK1-ALK fusion.

**Figure 3:**
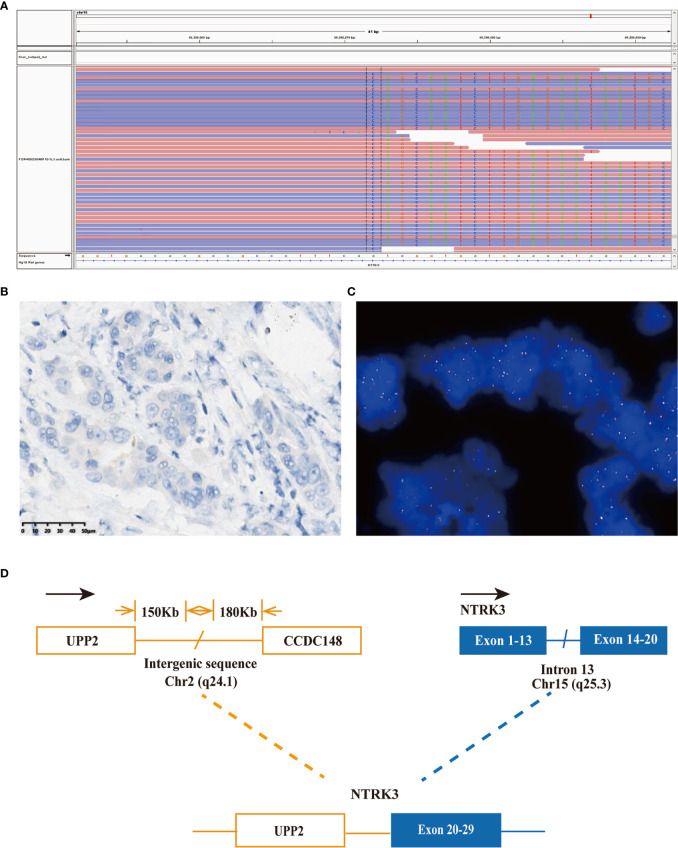
A novel UPP2-NTRK3 intergenic fusion was discovered in a PDCA patient. **(A)** Sequencing reads of NTRK3 is shown by the integrative genomics viewer. **(B)** Immunohistochemistry staining indicated expression of NTRK3; the specimen was stained by IHC with the VENTANA pan-TKR (EPR17341) antibody. **(C)** A split signal was observed with a frequency of 6% in the fluorescence *in situ* hybridization image using ZytoLight NTRK3 Break Apart FISH Probe. **(D)** Illustration of UPP2-NTRK3 intergenic fusion.

Considering the possibility of precision treatment, the surgical specimen was submitted to NGS panel analysis, which cover the whole exon regions of 539 cancer-related genes. The qualified DNA libraries were sequenced on Illumina NovaSeq6000 platform (Illumina, San Diego, CA, USA) and generate 150-bp paired-end reads. Base calls from Illumina NovaSeq6000 were conducted to FASTQ files. BWA-MEM (v.0.7.17) algorithm was then performed to align to the reference genome (hg19 GRCh37 of UCSC). SNVs/InDels were called and annotated *via* VarDict (v.1.5.7) (11) and InterVar (12), then the variants were filtered against the common SNPs in public database including 1,000 Genome Project and Exome Aggregation Consortium (ExAC) Browser28 (v.0.3). Fusions were analyzed by factera (v1.4.4) (13). The NGS results suggested that KRAS wild type. Beyond that, a somatic novel KANK1-ALK and UPP2 intergenic NTRK3 fusion were identified in this patient. The mutation profile revealed that these two fusions retained ALK/NTRK3 kinase domain (**Figure 2**). Fusions of KANK1-ALK and UPP2-NTRK3 with a frequency of 9.87% and 4.01% were detected in this patient respectively. It is worth mentioning that KANK1-ALK and UPP2-NTRK3 fusions have not been reported in any other solid tumor. What is more, both FISH and immunohistochemistry (IHC) results showed the KANK-ALK fusion could generate active protein. However, the UPP2 intergenic NTRK3 fusion showed the negative result in FISH and IHC (**Figure 3**), probably because complex rearrangements with intergenic breakpoints made transcription outcomes difficult to predict and confound detection (10). Interestingly, a BRAC1 p.Q12 germline pathogenetic mutation was also identified in this patient, which provided this patient with more potential options in druggable therapy.

## Discussion

ALK is one of the membrane-bound receptor tyrosine kinases, which consist of an extracellular ligand-binding domain, a single transmembrane domain, and a cytoplasmic tyrosine kinase domain. Previous comprehensive genomic profiling of 3,426 PDACs shows only five patients harbored an ALK fusion gene. Fusion partners were analogous to NSCLC, and most of them were EML4-ALK in PDAC (14). The NTRK gene family, NTRK1, NTRK2, and NTRK3 produce TRKA, TRKB, and TRKC proteins, respectively. All three TRK proteins can become targets of structural rearrangement caused by an NTRK gene fusion that results in a chimeric TRK fusion protein that drives uninterrupted downstream signaling. Moreover, the US FDA approved larotrectinib in November 2018 and entrectinib in August 2019, respectively; all of them got a great response for solid tumors harboring oncogenic NTRK fusions (15). As a result, these fusions have emerged as important targets for cancer therapy. In addition, as a result of the POLO trial study, the US FDA approved olaparib as a maintenance treatment for germline BRCA-mutated advanced PDAC that has not progressed on platinum-based chemotherapy. Therefore, PDAC patients with these driver mutations are likely to benefit from target therapy.

The most common type of malignant tumor of the pancreas is PDAC (16). Surgical resection is the only curative treatment, and it significantly improves the 5-year survival rate to 20%–30%. However, only <20% of all patients are eligible for resection as most patients are diagnosed at an advanced stage when there is metastasis (17). Poor prognosis is caused by the rapid progression, early metastasis, and lack of typical clinical presentation or sensitive screening methods for early-stage pancreatic cancer. Current therapeutic regimens, including FOLFIRINOX, gemcitabine plus nab-paclitaxel, and liposomal irinotecan with fluorouracil, have limited efficacy, with an incremental survival benefit of only a few months in unselected patients, underscore the need for novel treatment strategies. Molecular profiling of metastatic pancreatic cancer has become a routine practice. The NGS method has expanded over 90 different fusion partners, and newly identified potential KANK1-ALK and UPP2-NTRK3 fusion, and offers multitargetable opportunities to this patient (18). Gene fusion can be detected using various testing methods, including NGS, IHC, and FISH. Interestingly, both results of IHC and FISH in KNAK1-ALK fusion are positive, while the UPP2-NTRK3 intergenic fusion got the opposite result from IHC and FISH. A recent study revealed that intergenic breakpoint fusions, in which one or both genomic breakpoints localize to intergenic regions, may not produce chimeric fusion protein which would result in NGS and IHC or FISH results inconsistent (10). Compared with the significant false-positive data that occurred on the traditional test approaches, NGS, regardless of DNA or RNA based, is a forceful tool for detecting a low abundance of gene fusions in practice. Due to the potential limitation of DNA-based sequencing, RNA-based sequencing, or IHC, FISH assay may offer a more precision fusion detection (4).

Based on the result of NGS and clinical practice, and the POLO study evidence, the patient was started on olaparib 300 mg twice daily as maintenance treatment. In a previous comprehensive genomic profiling of 3,170 PDACs, only five cases identified ALK fusion. Four of five patients were treated with an ALK inhibitor, and three of these patients demonstrated stable disease, radiographic response, and/or normalization of serum CA 19-9 (6). In the light of NGS and validation results, we found a robust KNAK1-ALK fusion in this patient who may also be considered for ALK inhibitors in the future. In a previous case report, patients with NTRK fusion that simultaneously verified by NGS, FISH, and IHC are well tolerated to NTRK inhibitor (19). In this case, the UPP2 intergenic NTRK3 fusion in NGS and traditional method got an inconsistent result, which gives rise to the use of NTRK inhibitors that should be considered carefully. Thus, the limitation of this case is that there was no RNA-based hybrid capture revalidation due to the inadequate specimen, and the follow-up time is still relatively short. In addition, prospective clinical studies are still needed to explore the clinical prognosis of fusion-positive PDAC.

Herein, we firstly presented a case about pancreatic ductal adenocarcinoma patient concurrent to targetable rare somatic novel KANK1-ALK, UPP2-NTRK3 fusion, and pathogenetic germline BRCA mutation. To the best of our knowledge, KANK1-ALK and UPP2 intergenic NTRK3 fusions, as two novel fusions, have not been revealed in any solid tumor. In this case, concurrent actionable mutations provide more treatment opportunities in the future for the patient.

## Concluding Remarks

This case provides a new reference for understanding ALK and NTRK fusion mutations, discovers new molecular characteristics of PDAC patients, and provides the possibility for the future application of ALK-TKIs in PDAC. To improve the management of PDAC in an era of precision medicine, it is important to identify subsets of patients who can benefit from targeted therapies. The opportunity to implement precision medicine in oncology has grown in parallel with the increasing use of NGS. This case is an excellent example.

## Data Availability Statement

The original contributions presented in the study are included in the article/supplementary files, further inquiries can be directed to the corresponding author.

## Ethics Statement

Ethical approval was not provided for this study on human participants because The authors’ institution does not require ethical approval for the publication of a single case report. Written informed consent was obtained from the patient. The patients/participants provided their written informed consent to participate in this study. Written informed consent was obtained from the individual(s), and minor(s)’ legal guardian/next of kin, for the publication of any potentially identifiable images or data included in this article.

## Author Contributions

FM, YT, and QD prepared the manuscript and the literature search. HL reviewed and edited the manuscript. FM treated and observed the patient. LL performed the histopathological and immunohistochemical examinations. All authors contributed to the article and approved the submitted version.

## Conflict of Interest

Authors YT and QD were employed by companies Simcere Medical Laboratory Science Co., Ltd and Jiangsu Simcere Diagnostics Co., Ltd.

The remaining authors declare that the research was conducted in the absence of any commercial or financial relationships that could be construed as a potential conflict of interest.

## Publisher’s Note

All claims expressed in this article are solely those of the authors and do not necessarily represent those of their affiliated organizations, or those of the publisher, the editors and the reviewers. Any product that may be evaluated in this article, or claim that may be made by its manufacturer, is not guaranteed or endorsed by the publisher.

## Abbreviations

PDAC, pancreatic ductal adenocarcinoma; NGS, next-generation sequencing; FISH, fluorescence *in situ* hybridization; IHC, immunohistochemistry; NTRK, neurotrophic tropomyosin receptor kinases; ALK, anaplastic lymphoma kinase.
